# IQGAP3 Overexpression Correlates With Poor Prognosis and Radiation Therapy Resistance in Breast Cancer

**DOI:** 10.3389/fphar.2020.584450

**Published:** 2021-01-14

**Authors:** Xin Hua, Zhi-Qing Long, Ling Guo, Wen Wen, Xin Huang, Wen-Wen Zhang, Huan-Xin Lin

**Affiliations:** ^1^ State Key Laboratory of Oncology in South China, Collaborative Innovation Center for Cancer Medicine, Sun Yat-Sen University Cancer Center, Guangzhou, China; ^2^ Department of Radiotherapy, Sun Yat-Sen University Cancer Center, Guangzhou, China; ^3^ Department of Nasopharyngeal Carcinoma, Sun Yat-Sen University Cancer Center, Guangzhou, China

**Keywords:** breast cancer, IQGAP3, prognosis, radiation therapy, resistance

## Abstract

**Background:** IQ motif-containing GTPase activating protein 3 (IQGAP3), the latest identified member of the IQGAP family, may act as a crucial factor in cancer development and progression; however, its clinical value in breast cancer remains unestablished. We explored the correlation between IQGAP3 expression profile and the clinicopathological features in breast cancer.

**Methods:** IQGAP3 mRNA and protein levels were detected in breast cancer cell lines and tumor tissues by real-time PCR and western blotting and compared to the normal control groups. Protein expression of IQGAP3 was also evaluated immunohistochemically in archived paraffin-embedded specimens from 257 breast cancer patients, and the associations between IQGAP3 expression level, clinical characteristics, and prognosis were analyzed. We assessed the relationship between IQGAP3 expression and sensitivity to radiation therapy which was determined by subgroup analysis.

**Results:** IQGAP3 was significantly upregulated in breast cancer cell lines and human tumor tissues at both the mRNA and protein level compared to controls. Additionally, high levels of IQGAP3 expression were detected in 110/257 (42.8%) of archived paraffin-embedded breast cancer specimens. High IQGAP3 expression level was significantly related to clinical stage (*p* = 0.001), T category (*p* = 0.002), N category (*p* = 0.001), locoregional recurrence (*p* = 0.002), distant metastasis (*p* = 0.001), and vital status (*p* = 0.001). Univariate and multivariate statistical analysis showed that IQGAP3 expression was an independent prognostic factor among all 257 breast cancer patients in our cohort (*p* = 0.003, *p* = 0.001). Subgroup analysis revealed IQGAP3 expression correlated with radioresistance and was also an independent predictor of radiotherapy outcome.

**Conclusion:** Our findings suggest that high IQGAP3 expression predicts poor prognosis and radioresistance in breast cancer. Therefore, IQGAP3 may be a reliable prognostic biomarker in breast cancer and could be used to identify patients who may benefit from radiotherapy.

## Introduction

Breast cancer is the most frequent malignancy in women and the second leading cause of cancer-related deaths worldwide (Siegel et al., 2015). Radiotherapy (RT) is an indispensable part of the systemic therapeutic regimen for breast cancer, yet locoregional recurrence and distant metastasis remain key problems, resulting in poor survival (Gerber et al., 2010). Locoregional recurrence results from the presence or evolution of radioresistant tumor cells for which standard fractionated RT doses are sublethal (Speers et al., 2015). Currently, there is a dearth of clinically available predictive biomarkers to indicate the optimal radiation dosing in breast cancer, which leads to suboptimal treatment of these patients (Coates et al., 2015). Therefore, biomarkers associated with disease prognosis and RT sensitivity are required to optimize RT treatment plans and improve outcomes among breast cancer patients.

IQ motif-containing GTPase activating protein 3 (IQGAP3), the latest identified member of the IQGAP family, is an evolutionarily conserved GTPase-activating protein (Wang et al., 2007) and a hotspot for gene amplification in tumors. IQGAPs comprise five conserved domains: an IQ domain with four IQ motifs (IQ), a poly-proline protein-protein domain (WW), a calponin homology domain (CHD), a RasGAP-related domain (GRD), and a carboxy-terminal domain (RasGAPC) (Briggs and Sacks, 2003). Multiple proteins interact with these domains to regulate diverse cellular processes, including cell migration, cytokinesis, vesicle trafficking, cell proliferation, intracellular signaling, and cytoskeletal dynamics (Nojima et al., 2008).

Overexpression of IQGAP3 has been observed in lung, liver, pancreatic, and gastric cancer (Yang et al., 2014; Xu et al., 2016; Oue et al., 2017; Shi et al., 2017). Recently, IQGAP3 expression was found to be related to clinical stage and was an independent prognostic classifier of gastric cancer patients. Additionally, IQGAP3 knockdown was shown to reduce the number and size of the spheres formed by a gastric cancer cell line (MKN-74) and inhibited the phosphorylation of Akt and Erk1/2 (11). In hepatocellular carcinoma, IQGAP3 was reported to function as an important regulator of epithelial-mesenchymal transition (EMT) and metastasis by activating the transforming growth factor (TGF)-β signaling pathway (Shi et al., 2017). Regarding breast cancer, IQGAP3 knockdown inhibited cell proliferation and invasion in two breast carcinoma cell-lines (Hu et al., 2016). These reports provide some clues about IQGAP3 expression changes in several cancer types and the role of IQGAP3 in tumor development. However, the correlation between IQGAP3 expression and prognosis or RT sensitivity in breast cancer remained unclear.

Therefore, we investigated the expression pattern of IQGAP3 in breast cancer cell lines compared to control cell lines, as well as in patient tissues and matched adjacent normal tissues. We also analyzed the association of IQGAP3 protein expression with the survival outcomes and RT sensitivity in breast cancer patient cases.

## Materials and Methods

### Microarray Data

We performed integrative analyses on the Cancer Genome Atlas (TCGA) data for Breast Invasive Carcinoma-BRCA (Ye et al., 2016). mRNA expression data [mRNA fragments per kilobase transcript per million mapped reads (FPKM)] and matched clinical metadata (n = 1,208, 113 normal breast samples and 1,095 breast cancer samples) were acquired from the TCGA data portal https://portal.gdc.cancer.gov/projects/TCGA-BRCA. The FPKM data were first transformed into transcripts per million data for better comparison and edgeR package was used to normalize gene expression, and then the IQGAP3 expression value was extracted (Lv et al., 2019). Breast cancer patients who received RT were then divided into a radioresistant group (n = 115, who showed disease progression via locoregional recurrence) and a radiosensitive group (n = 600, without disease progression) according to their response to RT treatment.

### Cell Lines

Breast cancer cell lines, including MCF-10A, ZR-75-1, SK-BR-3, MDA-MB-468, MDA-MB-453, MCF-7, BT-474, MDA-MB-231, BT-549, HCC1937, SUM159PT, Hs-578T, and ZR-75-30, were cultured in DMEM medium (Gibco, Grand Island, NY) supplemented with 10% fetal bovine serum (FBS; HyClone, Logan, UT).

### Patients and Tissue Specimens

A total of 257 paraffin-embedded breast cancer tissue samples with histologically confirmed invasive carcinoma of no-specific-type breast cancer were collected from 2006 to 2008 at Sun Yat-sen University Cancer Center (SYSUCC), Guangzhou, China. In addition, six paired breast cancer and adjacent normal tissues were collected from patients who had undergone surgery from 2017 to 2018 at our center. All samples were frozen and stored in liquid nitrogen until further use. All patients received surgery and 201 (78.2%) received RT after surgery. The recommended indications for postoperative RT were involvement of ≥4 axillary nodes, primary tumor ≥5 cm in size, post-breast-conserving surgery, positive surgical margins, the involvement of internal mammary node (in selected cases, n = 3), and the involvement of one to three axillary nodes (in selected cases, n = 15). Clinicopathological classification and staging were determined according to the criteria of the American Joint Committee on Cancer (AJCC 2010; seventh edition). This study was approved by the Clinical Research Ethics Committee of SYSUCC, and written informed consent was obtained from each patient.

### PCR

We used quantitative real-time PCR (qRT-PCR) to evaluate mRNA expression of IQGAP3 in 12 breast cancer cell lines and a control cell line (MCF-10A), as well as in six tumor tissues and adjacent normal control tissues obtained from breast cancer patients. Total RNA samples from cell lines and freshly frozen tissues were isolated using TRIzol reagent (Invitrogen, Carlsbad, CA, USA) following the manufacturer’s recommendations. qRT-PCR was performed according to previously described standard methods, using GADPH as a control (Liu et al., 2017). All primers were designed using Primer Express version 2.0 software (Applied Biosystems, Foster City, CA, USA) as follows: IQGAP3 forward: 5′-ATG​AGC​AGA​GGC​GGC​AGA​AT-3′, reverse: 5′-GAA​CCA​CGG​AGG​GTG​CAA​AA-3′, GAPDH forward: 5′-GTC​TCC​TCT​GAC​TTC​AAC​AGC​G-3′, and reverse: 5′-ACC​ACC​CTG​TTG​CTG​TAG​CCA​A-3′. IQGAP3 expression levels were normalized to the geometric mean of GAPDH and calculated using 2^−[(Ct of IQGAP3)−(Ct of GAPDH)]^, where Ct represents the threshold cycle for each transcript. Each experiment was performed in triplicate.

### Western Blot

Western blot analysis was used to evaluate IQGAP3 protein expression in all 12 breast cancer cell lines and the control cell line (MCF-10A), as well as in six tumor tissue samples and adjacent normal tissues from breast cancer patients. Western blotting was carried out as described previously (Li et al., 2008) using the anti-IQGAP3 antibody (Abcam, Cambridge, MA). The membranes were stripped and reprobed with an anti-α-tubulin antibody (Sigma, Saint Louis, MI) as a loading control.

### Immunohistochemistry

Immunohistochemistry (IHC) and quantification of IQGAP3 expression were performed by two independent pathologists, as previously described (Song et al., 2012) using an anti-IQGAP3 antibody (1:1,000; Sigma, Saint Louis, MI). The percentage of cancer cells was scored as 1 (<10%), 2 (10–50%), 3 (50–75%), or 4 (>75%), and the staining intensity was sorted into four grades: 0 (no staining), 1 (weak staining, light yellow), 2 (moderate staining, yellow brown), and 3 (intense staining, brown). The scores for the staining intensity and proportion in each section were multiplied. The best cutoff value for IQGAP3 was defined by receiver operating curve (ROC) analysis with respect to overall survival: a staining score≥6 was classified as high expression and a score≤4 as low IQGAP3 expression.

### Gene Set Enrichment Analysis

Gene set enrichment analysis (GSEA, http://software.broadinstitute.org/gsea/index.jsp) was used to predict potential hallmarks using transcriptional sequences in the TCGA database. GSEA was performed on the gene list and ranked according to a moderated t-statistics (Ritchie et al., 2015), comparing patients with high and low IQGAP3 expression. A permutation test (of 1,000 times) was used to identify the significantly changed pathways (Subramanian et al., 2005).

### Statistical Analysis

All statistical analyses were conducted using SPSS (version 20.0; IBM Corporation, Armonk, NY, USA), and the survival curves were drawn using the GraphPad Prism 6.0 Software. The Pearson’s χ^2^ tests or Fisher exact tests were used to analyze the associations between IQGAP3 expression and clinicopathological features. The Kaplan-Meier method and log-rank test were used to calculate and compare the curve survival curves. Multivariate analysis was performed via a Cox’s proportional hazards model. A two-sided *p*-value of <0.05 was considered statistically significant.

## Results

### IQGAP3 Expression Is Elevated in Breast Cancer Cell Lines

**FIGURE 1:**
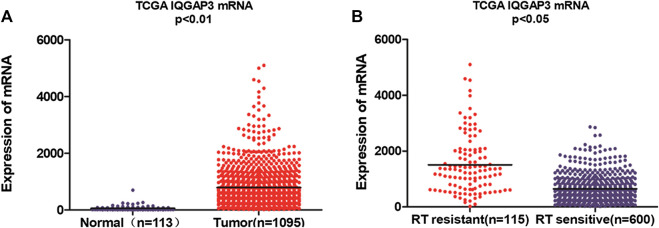
Microarray data reveals IQGAP3 is upregulated in breast cancer patients and in cases that are resistant to radiotherapy (RT). **(A)** Expression of IQGAP3 in TCGA (breast invasive carcinoma) tumor and normal tissue data (Mann–Whitney test; *p* < 0.01). **(B)** Expression of IQGAP3 in TCGA (breast invasive carcinoma) including RT-resistant and RT-sensitive cases (Mann–Whitney test; *p* < 0.05).

**FIGURE 2:**
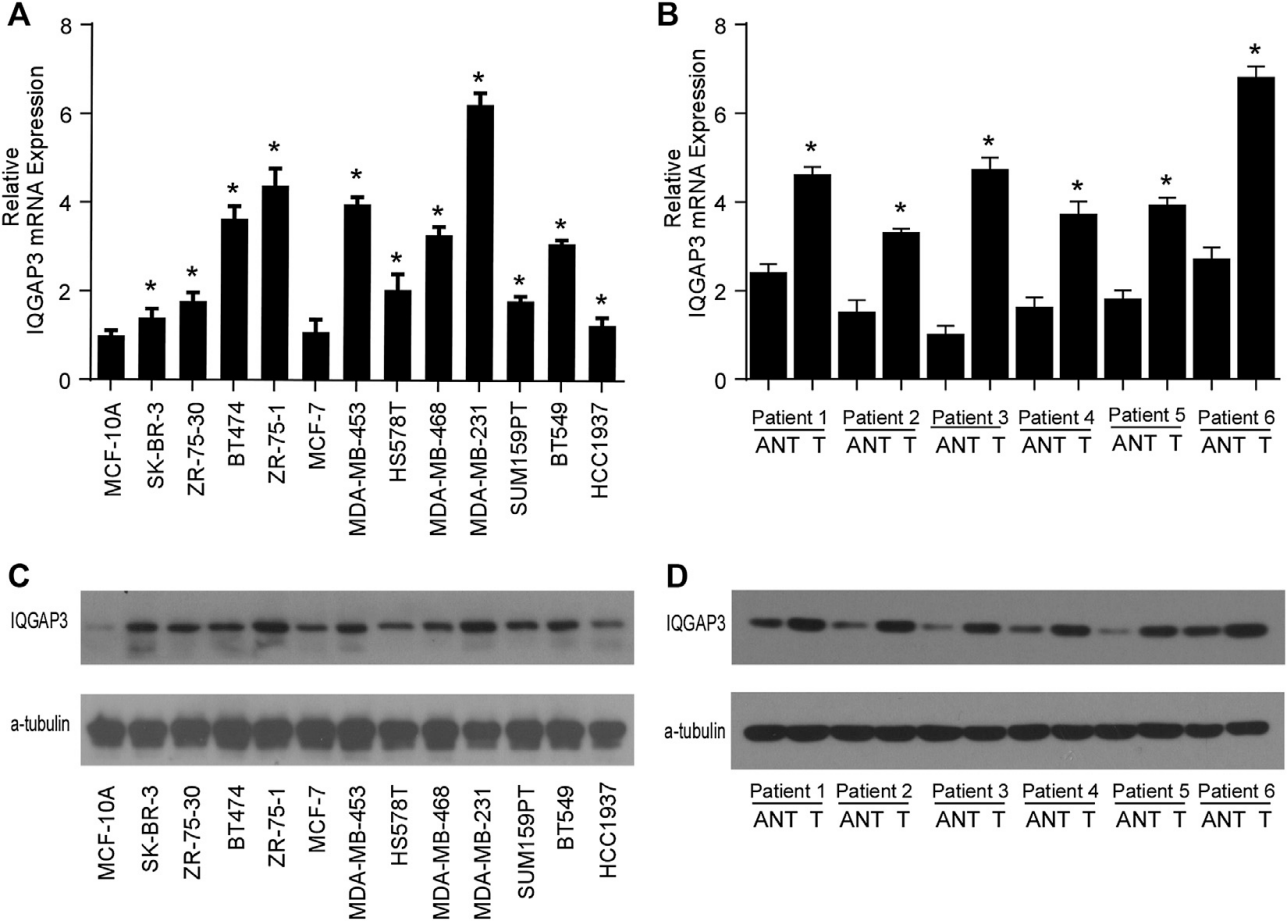
IQGAP3 is upregulated in breast cancer cell lines and tissues. **(A)** Quantitative real-time PCR analysis of IQGAP3 mRNA expression in MCF-10A immortalized breast epithelial cells and twelve cultured breast cancer cell lines. GAPDH was used as a loading control. *p < 0.05. **(B)** Real-time PCR analysis of IQGAP3 mRNA expression in six paired breast cancer tumor tissues and adjacent normal tissues (ANT). GAPDH was used as a loading control. *p < 0.05. **(C)** Western blotting analysis of IQGAP3 protein expression in MCF-10A immortalized breast epithelial cells and twelve cultured breast cancer cell lines. α-tubulin was used as a loading control. **(D)** Western blotting analysis of IQGAP3 protein expression in six paired breast cancer tumor tissues and adjacent normal tissues (ANT). α-tubulin was used as a loading control.

We found IQGAP3 mRNA levels were increased in tumor samples compared with normal tissues by analyzing the publicly available microarray TCGA data for breast cancer (Figure 1A). Additionally, we found IQGAP3 expression was elevated at both the mRNA and protein level in all 12 breast cancer cell lines compared with the MCF-10A control cell line (Figure 2A). Higher IQGAP3 expression was also detected at the mRNA and protein level in breast cancer tissues compared to adjacent normal tissues from six different patients (Figure 2B). Together, these results indicated IQGAP3 is overexpressed in breast cancer cell lines and tissues.

### IQGAP3 Overexpression Correlates With the Clinicopathological Features of Breast Cancer

**FIGURE 3:**
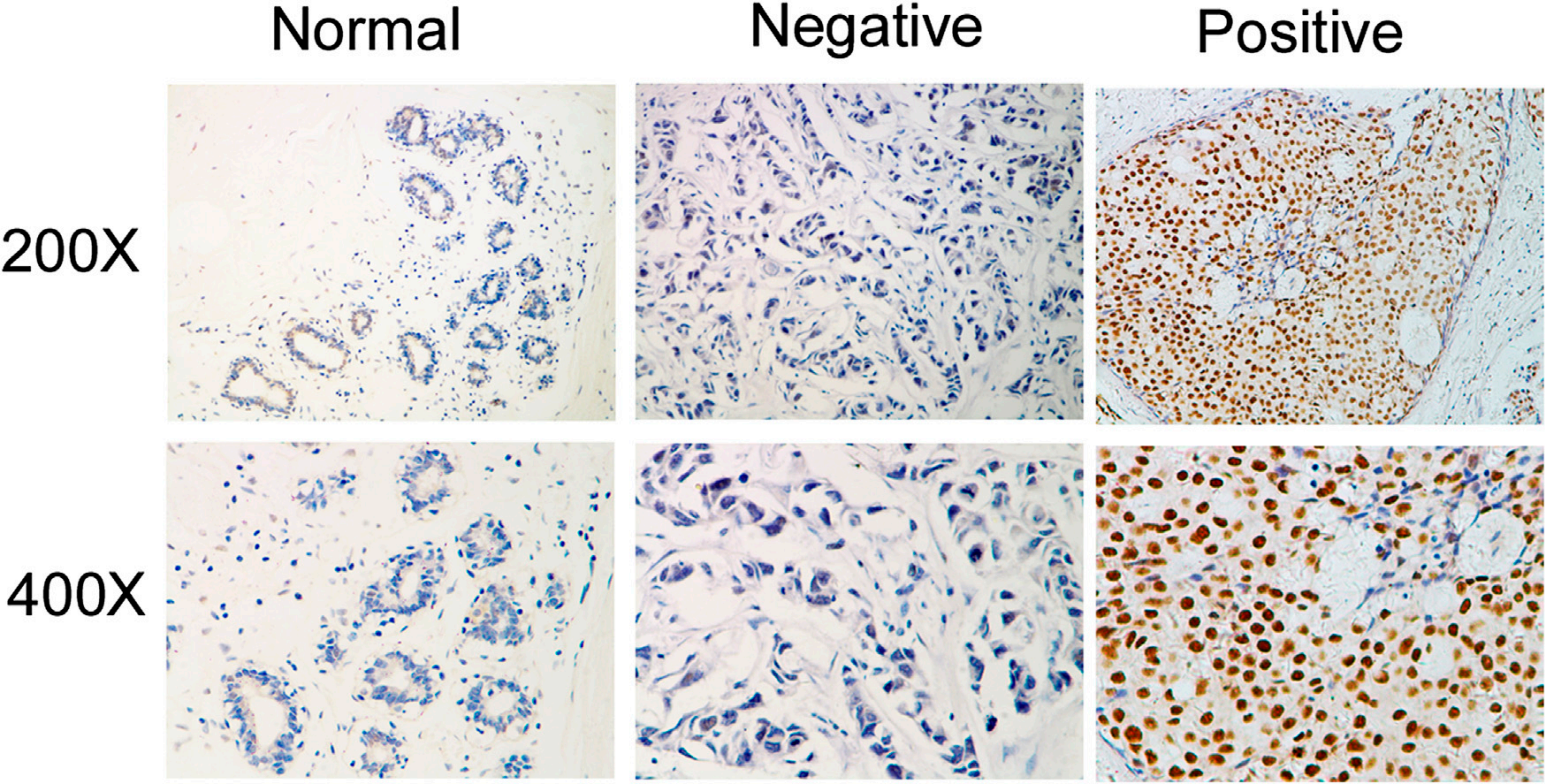
Immunohistochemical detection of IQGAP3 expression in paraffin-embedded breast cancer tissues. Representative images of immunohistochemical staining for IQGAP3 in normal breast tissues (controls) and breast tumor tissues are shown.

Next, we investigated whether IQGAP3 overexpression levels in 257 cases of breast cancer specimens (detected via IHC) were associated with patients’ clinicopathological features. Among the 257 breast cancer cases, 21 were in stage Ⅰ (8.2%), 32 were in stage Ⅱ (12.5%), and 204 were in stage Ⅲ (79.4%). A total of 110 samples (42.8%) had a higher level of IQGAP3 protein level expression (staining was strongly positive). A lower expression level (staining was weakly positive or negative) was found in 147 samples (57.2%, Table 1). Positive IQGAP3 staining was observed mainly in the cancer cell nuclei (Figure 3). IQGAP3 overexpression significantly correlated with the following characteristics: clinical stage (*p* = 0.001), T category (*p* = 0.010), N category (*p* = 0.001), distant metastasis (*p* = 0.001), locoregional recurrence (*p* = 0.002), and vital status (*p* = 0.001; Table 1).

**TABLE 1:** Association between IQGAP3 expression and clinicopathological features of breast cancer (n = 257).

Feature	Total (n = 257)	IQGAP3		P
		Low expression	High expression	
Age (y)				
≥45	138 (53.7%)	78 (56.5%)	60 (43.5%)	0.899
<45	119 (46.3%)	69 (58.0%)	50 (42.0%)	
T classification				
1	53 (20.6%)	34 (64.2%)	19 (35.8%)	**0.010**
2	132 (51.4%)	83 (62.9%)	49 (37.1%)	
3	42 (16.3%)	20 (47.6%)	22 (52.4%)	
4	30 (11.7%)	10 (33.3%)	20 (66.7%)	
N classification				
0	38 (14.8%)	27 (71.1%)	11 (28.9%)	**0.001**
1	28 (10.9%)	21 (75.0%)	7 (25.0%)	
2	102 (39.7%)	65 (63.7%)	37 (36.3%)	
3	89 (34.6%)	34 (38.2%)	55 (61.8%)	
Clinical stage				
I	21 (8.2%)	14 (66.7%)	7 (33.3%)	**0.001**
II	32 (12.5%)	27 (84.4%)	5 (15.6%)	
III	204 (79.4%)	106 (52.0%)	98 (48.0%)	
Locoregional recurrence				
yes	23	6	17	**0.002**
no	234	141	93	
Distant metastasis				
Yes	72	18	54	**0.001**
No.	185	129	56	
Vital status				
Alive	203 (79.0%)	134 (66.0%)	69 (34.0%)	**0.001**
Dead	54 (21.0%)	13 (24.1%)	41 (75.9%)	
Treatment				
Radiotherapy	201 (78.2%)	114 (56.7%)	87 (43.2%)	0.879
Nonradiotherapy	56 (21.8%)	33 (58.9%)	23 (41.1%)	

P: *t* test with two independent samples.

**TABLE 2:** Association of clinicopathological features with overall survival and progression-free survival in breast cancer patients (n = 257).

Feature	Univariate analysis	*P*	Multivariate cox regression analysis	*P*
	Regression coefficient (SE)		Hazard ratio (95% CI)	
OS					
Age (y)					
≥45 vs. < 45	0.018 (0.014)	0.197	—	—
T stage					
T 1 vs. 2 vs. 3 vs. 4	0.665 (0.143)	**0.001**	1.823 (1.346–2.470)	**0.001**
N stage					
N 0 vs. 1 vs. 2	0.984 (0.211)	**0.001**	2.074 (1.395–3.084)	**0.001**
Estrogen receptor					
ER (−) vs. (+)	−1.514 (0.309)	**0.001**	0.206 (0.105–0.404)	**0.001**
Progesterone receptor					
PR (−) vs. (+)	−0.810 (0.270)	**0.003**	1.287 (0.712–2.326)	0.403
Human epidermal growth factor receptor type 2					
Her2 (−) vs. (+)	0.382 (0.249)	0.125	—	—
IQGAP3 expression					
Low vs. high	1.606 (0.319)	**0.001**	2.723 (1.419–5.224)	**0.003**
PFS					
Age (y)					
≥45 vs. < 45	0.020 (0.011)	0.0073	—	—
T stage					
T 1 vs. 2 vs. 3 vs. 4	0.585 (0.118)	**0.001**	1.592 (1.238–2.047)	**0.001**
N stage					
N 0 vs. 1 vs. 2	0.868 (0.163)	**0.001**	1.866 (1.377–2.528)	**0.001**
ER					
ER (−) vs. (+)	−1.315 (0.245)	**0.001**	0.314 (0.177–0.559)	**0.001**
PR					
PR (−) vs. (+)	−0.882 (0.226)	**0.001**	1.023 (0.600–1.746)	0.933
Her2					
Her2 (−) vs. (+)	0.467 (0.205)	**0.023**	1.248 (0.750–2.076)	0.393
IQGAP3 expression					
Low vs. high	1.673 (0.265)	**0.001**	3.160 (1.822–5.482)	**0.001**

### High IQGAP3 Expression Is Significantly Associated With Poor Prognosis in Breast Cancer

**FIGURE 4:**
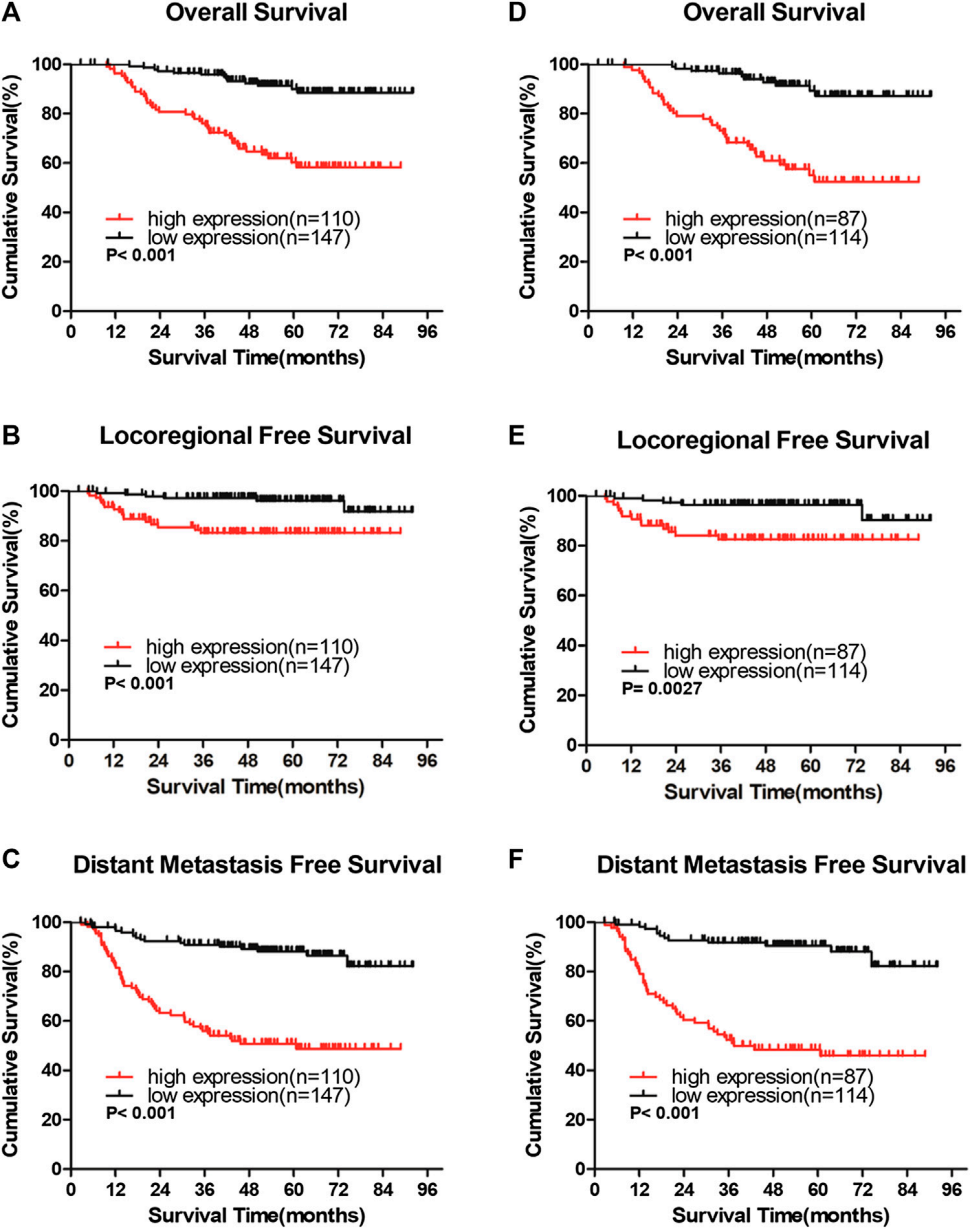
IQGAP3 protein expression is associated with clinical outcomes in the whole cohort of breast cancer cases and in the radiotherapy (RT) subgroup **(A and B,C)**. Kaplan–Meier overall survival **(A)**, locoregional recurrence-free survival **(B)**, and distant metastasis-free survival **(C)** curves for all 257 patients with breast cancer stratified by high IQGAP3 expression (n = 110) vs. low IQGAP3 expression (n = 147) **(D and E,F)**. Kaplan–Meier overall survival **(D)**, locoregional recurrence-free survival **(E)**, and distant metastasis free survival **(F)** curves for RT subgroup of 201 patients stratified by high IQGAP3 expression (n = 87) vs. low IQGAP3 expression (n = 114). *p* values were calculated using the log-rank test.

In the entire cohort, the 5-year overall survival (OS), locoregional recurrence-free survival (LRFS), and distant metastasis-free survival (DMFS) rates were as follows: 76.9%, 90.7%, and 71.9%, respectively. The cumulative 5-year OS, LRFS, and DMFS rates for patients with high IQGAP3 expression were 58.3%, 83.2%, and 50.8%, respectively, compared with 89.9%, 96.1%, and 88.2%, respectively, for patients with low IQGAP3 expression (*p* = 0.001, Figure 4A; *p* = 0.001, Figure 4B; and *p* = 0.001, Figure 4C).

We also evaluated the prognostic, predictive value of IQGAP3 overexpression in the RT subgroup (n = 201). IQGAP3 overexpression significantly correlated with poor OS, LRFS, and DMFS in patients who had undergone RT (*p* = 0.001, Figure 4D; *p* = 0.003, Figure 4E; and *p* = 0.001, Figure 4F). This data demonstrates that breast cancer patients with high levels of IQGAP3 show poor survival even after RT.

### IQGAP3 Overexpression Is an Independent Negative Prognostic Factor in Breast Cancer

Univariate Cox regression analysis showed that the T category, N category, estrogen receptor (ER) status, progesterone receptor (PR) status, and IQGAP3 expression were significantly associated with survival in breast cancer patients. Multivariate survival analysis also indicated IQGAP3 expression was indeed an independent prognostic factor for OS and progression-free survival (PFS; *p* = 0.003 and *p* = 0.001, respectively; Table 2) in the whole cohort breast cancer patients (n = 257). Multivariate survival analysis in the RT subgroup (n = 201) showed that IQGAP3 expression remained an independent prognostic factor for OS and PFS (*p* = 0.002 and *p* = 0.001, respectively; Table 3). These results suggest that IQGAP3 may be an independent prognostic factor of breast cancer treatment outcome, especially for patients who have undergone RT.

**TABLE 3:** Association of clinicopathological features with overall survival and progression-free survival in breast cancer patients undergoing radiotherapy (n = 201).

Feature	Univariate analysis	*P*	Multivariate cox regression analysis	*P*
	Regression coefficient (SE)		Hazard ratio (95%CI)	
**OS**				
Age (y)				
≥45 vs. < 45	−0.002 (0.015)	0.909	—	—
T stage				
T 1 vs. 2 vs. 3 vs. 4	0.642 (0.160)	**0.001**	1.851 (1.322–2.593)	**0.001**
N stage				
N 0 vs. 1 vs. 2	0.863 (0.228)	**0.001**	1.755 (1.156–2.662)	**0.008**
Estrogen receptor				
ER (−) vs. (+)	−1.561 (0.345)	**0.001**	0.181 (0.085–0.385)	**0.001**
Progesterone receptor				
PR (−) vs. (+)	−0.852 (0.295)	**0.004**	1.315 (0.689–2.509)	0.406
Human epidermal growth factor receptor type 2				
Her2 (−) vs. (+)	0.227 (0.290)	0.433	-	-
IQGAP3 expression				
Low vs. high	1.745 (0.358)	**0.001**	3.159 (1.515–6.586)	**0.002**
**PFS**				
Age (y)				
≥45 vs. < 45	0.005 (0.013)	0.702	—	—
T stage				
T 1 vs. 2 vs. 3 vs. 4	0.558 (0.137)	**0.001**	1.611 (1.207–2.150)	**0.001**
N stage				
N 0 vs. 1 vs. 2	0.836 (0.191)	**0.001**	1.677 (1.183–2.377)	**0.004**
ER				
ER (−) vs. (+)	−1.386 (0.285)	**0.010**	0.293 (0.151–0.568)	**0.010**
PR				
PR (−) vs. (+)	−0.994 (0.258)	**0.028**	0.947 (0.515–1.739)	0.860
Her2				
Her2 (−) vs. (+)	0.462 (0.245)	**0.023**	1.216 (0.683–2.166)	0.506
IQGAP3 expression				
Low vs. high	1.867 (0.313)	**0.001**	3.813 (1.995–7.286)	**0.001**

**TABLE 4:** Association of clinicopathological features with radioresistance-free survival in breast cancer cases resistant to radiation therapy (n = 159).

Feature	Univariate analysis	*P*	Multivariate cox regression analysis	*P*
	Regression coefficient (SE)		Hazard ratio (95%CI)	
RRFS				
Age (y)				
≥45 vs. < 45	0.009 (0.023)	0.690	—	—
T stage				
T 1 vs. 2 vs. 3 vs. 4	0.612 (0.245)	**0.012**	1.613 (0.964–2.700)	0.069
N stage				
N 0 vs. 1 vs. 2	1.029 (0.364)	**0.005**	1.901 (0.973–3.715)	0.060
ER				
ER (−) vs. (+)	−1.370 (0.493)	**0.005**	0.445 (0.142–1.400)	0.166
PR				
PR (−) vs. (+)	−1.801 (0.520)	**0.001**	0.365 (0.107–1.240)	0.106
Her2				
Her2 (−) vs. (+)	0.429 (0.441)	0.331	-	-
IQGAP3 expression				
Low vs. high	1.831 (0.521)	**0.001**	3.321 (1.135–9.716)	**0.028**

### IQGAP3 Overexpression Significantly Correlates With Radioresistance in Breast Cancer

Breast cancer patients who received RT were divided according to their response to treatment into a radioresistant group (who showed disease progression via locoregional recurrence) and a radiosensitive group (without disease progression). We first analyzed the public microarray TCGA data of breast cancer and found IQGAP3 was overexpressed in radioresistant samples (n = 115) compared to radiosensitive samples (n = 600; Figure 1B).

**FIGURE 5:**
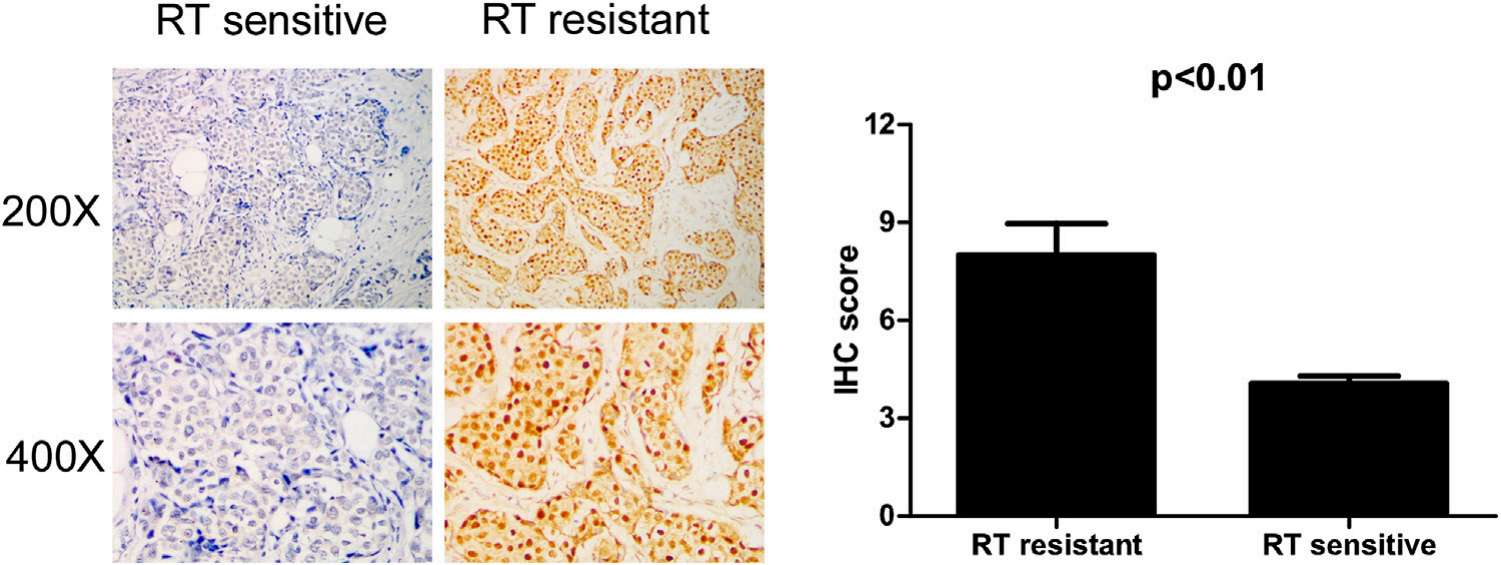
Immunohistochemical (IHC) detection of IQGAP3 expression in paraffin-embedded breast cancer tissues in the radiotherapy- (RT-) sensitive and RT-resistant subgroups. Left panel: representative images of IHC staining for IQGAP3 in the RT-resistant and RT-sensitive tissues. Right panel: average IHC score of IQGAP3 in the RT-resistant (n = 19) and RT-sensitive tissues (n = 140).

To confirm whether IQGAP3 is overexpressed in radioresistant breast cancer patients, we also examined IQGAP3 expression in 159 post-RT patients (radioresistant group n = 19; radiosensitive group n = 140) using IHC. IQGAP3 was overexpressed in radioresistant breast cancer patients compared to radiosensitive patients (Figure 5).

### IQGAP3 Overexpression Is an Independent Prognosis Factor for Radiation Therapy Outcome in Breast Cancer

Through subgroup analysis of the 159 post-RT cases, we discovered IQGAP3 overexpression was correlated with an obviously shorter radioresistance-free survival (RRFS). Univariate Cox regression analyses showed that IQGAP3 expression, T stage, N stage, ER, and PR were significant prognostic factors for RT outcome (*p* = 0.012, *p* = 0.005, *p* = 0.005, *p* = 0.001, and *p* = 0.001, respectively; Table 4). IQGAP3 overexpression remained an independent prognostic factor for shorter RRFS in multivariate analysis (HR: 3.321; 95% CI: 1.135–9.716; *p* = 0.028).

### IQGAP3 Overexpression May Promote DNA Damage Repair and Lead to Radiotherapy Resistance by Modulating the PI3K/AKT/mTOR Pathway

**FIGURE 6:**
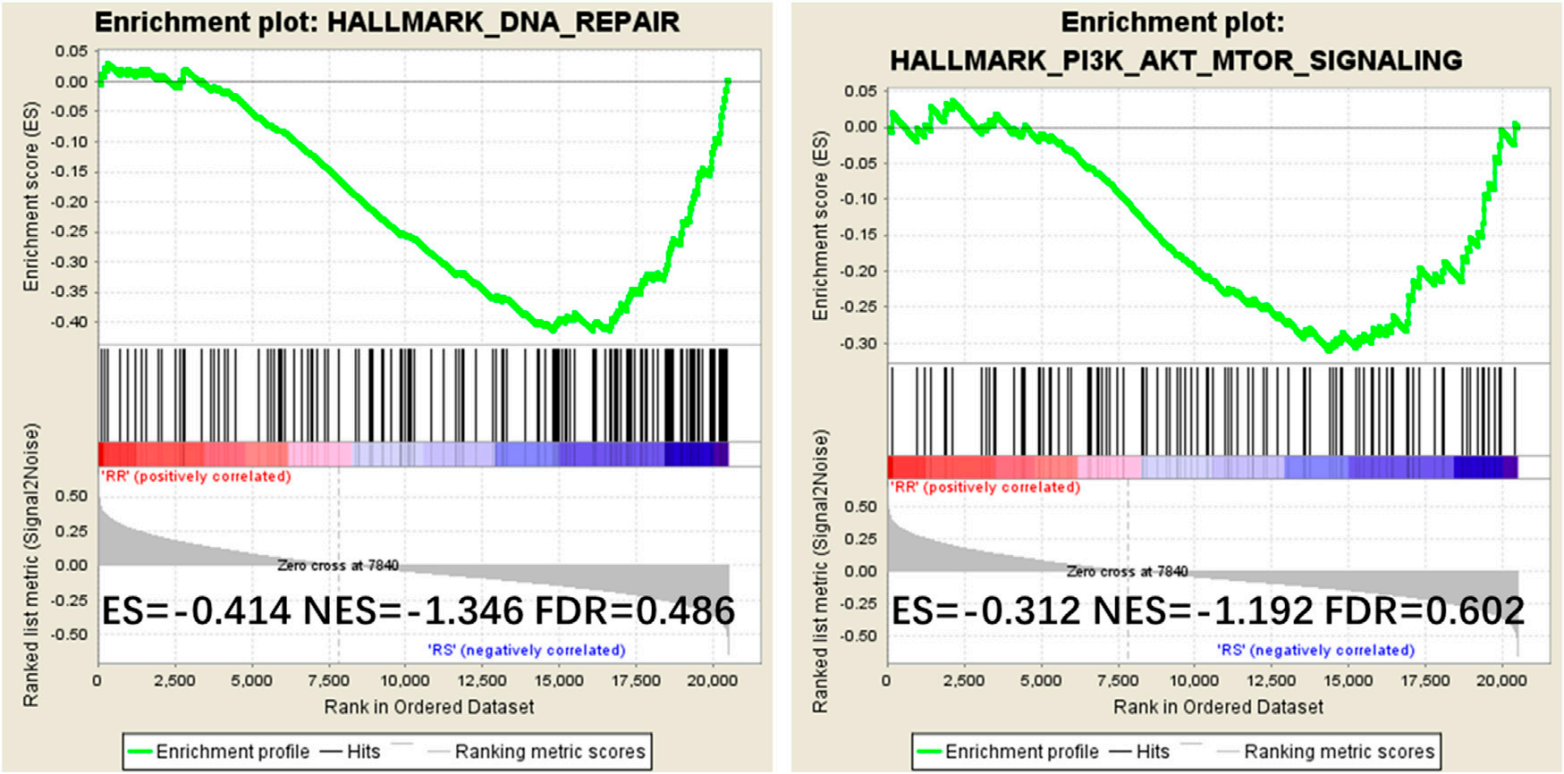
Gene Set Enrichment Analysis (GSEA) plots showing that IQGAP3 expression correlates positively with DNA repair gene signatures (HALLMARK_DNA_REPAIR) and PI3K signaling-activated gene signatures (HALLMARK_PI3K_AKT_MTOR_SIGNALING) in the published Cancer Genome Atlas (TCGA) breast invasive carcinoma gene expression profiles.

To examine the mechanism of IQGAP3 in the development of breast cancer radioresistance, we performed GSEA. We found IQGAP3 expression is positively correlated with DNA repair gene signatures (HALLMARK_DNA_REPAIR) and phosphatidylinositol-4,5-bisphosphate 3-kinase (PI3K) signaling-activated gene signatures (HALLMARK_PI3K_AKT_MTOR_SIGNALING) in TCGA published gene expression profiles (Figure 6).

## Discussion

Growing evidence suggests IQGAP3 is overexpressed in various tumors, including melanoma, pancreatic cancer, gastric cancer, bladder cancer, hepatocellular carcinoma, and breast cancer (Yang et al., 2014; Hu et al., 2016; Xu et al., 2016; Oue et al., 2017; Shi et al., 2017). IQGAP3 has also been suggested to help in screening and diagnosis by acting as a biomarker in hepatocellular carcinoma (Qian et al., 2016). Furthermore, recent studies showed that IQGAP3 overexpression accelerates cell proliferation and invasion in several tumors, indicating it may play a role in cancer progression (Yang et al., 2014; Hu et al., 2016; Kumar et al., 2017). However, its role in breast cancer remained vague until now. To the best of our knowledge, this is the first study to confirm an association between IQGAP3 expression and disease prognosis and RT sensitivity in breast cancer.

Previous studies have shown that IQGAP3 may promote and accelerate cancer development *in vitro*. For example, IQGAP3 knockdown inhibited proliferation and ERK activity in cultured epithelial cells (Nojima et al., 2008); IQGAP3 was also found to activate EGFR–ERK signaling and, thus, promote the metastasis of lung cancer cells (Yang et al., 2014). Furthermore, in two pancreatic cancer cell lines (BXPC-3 and SW1990), IQGAP3 knockdown inhibited cell proliferation, migration, and invasion and induced cell apoptosis (Xu et al., 2016). Moreover, silencing IQGAP3 was found to inhibit the proliferation, motility, and invasion of breast cancer cell lines (Hu et al., 2016). Consistent with this study involving breast cancer cell lines, the present research provides evidence that IQGAP3 expression may have important clinical significance in breast cancer.

We confirmed IQGAP3 was overexpressed both at the mRNA level (transcriptionally) and protein level (translationally) in breast cancer cell lines and human tumor samples compared to noncancerous breast epithelial cells and tissues. IQGAP3 overexpression significantly correlated with the following characteristics: gender, clinical stage, T category, N category, vital status, and distant metastasis. Moreover, patients with high IQGAP3 expression were more likely to exhibit locoregional recurrence and distant metastasis, indicating that IQGAP3 protein expression promotes the progression of breast cancer. We also found a significant association between high IQGAP3 expression and poorer 5-year OS, LRFS, and DMFS in both the entire cohort and the RT-treated subgroup. Together, this evidence suggests that IQGAP3 contributes to the development and progression of breast cancer.

Breast cancer recurrence ranges from 10 to 41%, depending on T status, N status, and tumor grade (Pan et al., 2017). Compared to other thoracic tumors, innate or acquired radioresistance leads to locoregional recurrence and results in treatment failure. Therefore, radioresistance represents a formidable clinical problem in the systematic treatment regimen of breast cancer. However, no reliable biomarkers are currently available to identify patients who may be radioresistant before undergoing RT in breast cancer. We found IQGAP3 was strongly positively associated with radioresistance, and high IQGAP3 protein expression significantly correlated with shorter LRFS and OS, even after RT treatment. Therefore, IQGAP3 may be a valuable biomarker to identify specific patients who need a more aggressive RT therapeutic regimen (such as a higher dose of radiation) to reduce locoregional recurrence and improve survival. Moreover, our multivariate analysis confirmed IQGAP3 was an independent prognostic factor for RRFS in the subgroup analysis of radiosensitive breast cancer cases. In conclusion, IQGAP3 may be a reliable novel predictive biomarker of radioresistance and poor survival in breast cancer patients following RT. It can be a potential marker to determine RT effect in the future, in addition to other traditional risk factors, like young age, vessel invasion, and a low number of examined axillary lymph nodes.

Tumor cellular exposure to radiation results in damage to DNA and other cellular structures, which then triggers a complex cascade of downstream response pathways in both the nucleus and cytoplasm, including DNA repair, cell cycle modulation, reactive oxygen species defense, cytokine production, and apoptosis (Pajic et al., 2018). In certain tumor cell subpopulations, these pathways can be innately biased towards a radioresistant, prosurvival phenotype (i.e., a phenotype with accelerated cell cycle arrest, reduced proliferation, more efficient or prolonged DNA repair, or dampened apoptotic signaling) (Gewirtz et al., 2009; Karar and Maity, 2009; Goldstein and Kastan, 2015). Indeed, IQGAP3 was found to bind to the Ras protein (Nojima et al., 2008), which plays a role in cell cycle arrest, DNA repair, proliferation, and antiapoptosis in human cancers (Simanshu et al., 2017). Studies have also shown that IQGAP3 can regulate certain signaling pathways and cellular functions (Hedman et al., 2015), including mitogen-activated protein kinase (MAPK) signaling, Ca2+/calmodulin signaling, cell–cell adhesion, β-catenin-mediated transcription, and microbial invasion.

To further examine the mechanism of IQGAP3 in the development of breast cancer radioresistance, we performed GSEA and found IQGAP3 expression positively correlates with DNA repair gene signatures (HALLMARK_DNA_REPAIR) and phosphatidylinositol-4,5-bisphosphate 3-kinase (PI3K) signaling-activated gene signatures (HALLMARK_PI3K_AKT_MTOR_SIGNALING) in published TCGA gene expression profiles. The PI3K signaling pathway is regulated by Ras; that is, the direct binding of Ras to the catalytic p110 subunit can directly activate PI3K. The PI3K pathway may contribute to the repair, regrowth, redistribution, and reoxidation of cells after RT. As per evidence, Fan et al. demonstrated that increased expression of PI3K in the breast cancer cell line MDA-DB-453 after RT not only protects cells from apoptosis but also significantly enhances their DNA repair ability (Fan et al., 2001). In addition, reducing PI3K signaling with a PI3K inhibitor (LY294002) after RT can lead to G2/M cell cycle arrest in a breast cell line MCF-7 (Shtivelman et al., 2002). Based on the above-mentioned evidence, we assume that high levels of IQGAP3, combined with Ras, may promote radioresistance in breast cancer by modulating the PI3K signaling pathway. Although many PI3K inhibitors are currently undergoing investigation in clinical trials, CAL-101 was the first PI3K inhibitor to be approved by the US Food and Drug Administration and the European Medicines Agency for the treatment of different types of leukemia in 2014 (Bendell et al., 2012; Shapiro et al., 2014).

There are some limitations to our study. First, it was a retrospective study, and the cohort size was not sufficiently large. Second, we lack direct evidence to support the role(s) of IQGAP3 in breast cancer progression and radioresistance. Therefore, further biochemical studies into the precise mechanism(s) of action of IQGAP3 are warranted.

## Conclusion

This study shows IQGAP3 is overexpressed in breast cancer cell lines and tissues and is associated with the clinicopathological features of the disease. Additionally, IQGAP3 overexpression correlates with radioresistance and significantly poorer prognosis. Therefore, IQGAP3 may be a reliable novel biomarker to provide personalized prognostication in breast cancer and could be used to identify patients who may benefit from more aggressive RT treatment to improve their survival.

## Data Availability Statement

The data and materials of this study have been included at RDD (http://www.researchdata.org.cn/) with the number of RDDB2019000551.

## Ethics Statement

The studies involving human participants were reviewed and approved by the Clinical Research Ethics Committee of SYSUCC. The patients/participants provided their written informed consent to participate in this study.

## Author Contributions

Conceptualization was done by W-WZ H-XL; methodology was conceptualized by XH and Z-QL; software was provided by XH Z-QL; validation was carried out by XH and WW; formal analysis was done by XH and H-XL; investigation was conducted by WW and W-WZ; resources were provided by LG, H-XL, and W-WZ; data curation was done by H-XL, WW, and Z-QL; writing (original draft preparation) was done by XH and Z-QL; writing (review and editing) was done by W-WZ and H-XL; visualization was carried out by LG and W-WZ; supervision was given by W-WZ and H-XL; W-WZ and H-XL was responsible for project administration; funding acquisition was carried out by LG, H-XL, and W-WZ; all authors have read and approved the paper.

## Funding

This work was partly supported by the National Natural Science Foundation of China (nos. 81773103, 81772877, and 81572848) and Natural Science Foundation of Guangdong Province (2017A030313617).

## Conflict of Interest

The authors declare that the research was conducted in the absence of any commercial or financial relationships that could be construed as a potential conflict of interest.
